# Infant frontal EEG asymmetry in relation with postnatal maternal depression and parenting behavior

**DOI:** 10.1038/tp.2017.28

**Published:** 2017-03-14

**Authors:** D J Wen, N N Soe, L W Sim, S Sanmugam, K Kwek, Y-S Chong, P D Gluckman, M J Meaney, A Rifkin-Graboi, A Qiu

**Affiliations:** 1 Department of Biomedical Engineering, Clinical Imaging Research Center, National University of Singapore, Singapore, Singapore; 2 Singapore Institute for Clinical Sciences, Singapore, Singapore; 3 KK Women's and Children's Hospital, Singapore, Singapore; 4 Department of Obstetrics & Gynaecology, Yong Loo Lin School of Medicine, National University of Singapore, Singapore, Singapore; 5 Ludmer Centre for Neuroinformatics and Mental Health, Douglas Mental Health University Institute, McGill University, Montréal, QC, Canada; 6 Sackler Program for Epigenetics & Psychobiology, McGill University, Montréal, QC, Canada

## Abstract

Right frontal electroencephalogram (EEG) asymmetry associates with negative affect and depressed mood, which, among children, are predicted by maternal depression and poor parenting. This study examined associations of maternal depression and maternal sensitivity with infant frontal EEG asymmetry based on 111 mother-6-month-infant dyads. There were no significant effects of postnatal maternal depression or maternal sensitivity, or their interaction, on infant EEG frontal asymmetry. However, in a subsample for which the infant spent at least 50% of his/her day time hours with his/her mother, both lower maternal sensitivity and higher maternal depression predicted greater relative right frontal EEG asymmetry. Our study further showed that greater relative right frontal EEG asymmetry of 6-month-old infants predicted their greater negative emotionality at 12 months of age. Our study suggested that among infants with sufficient postnatal maternal exposure, both maternal sensitivity and mental health are important influences on early brain development.

## Introduction

Maternal depression and poor parenting predict childhood behavioral and emotional difficulties.^1, 2, 3, 4^ Compared to children of non-depressed mothers, children of depressed mothers display more developmental problems,^5^ greater stress system dysregulation,^6^ more negative affect,^7^ poorer affect regulation,^8^ more behavior problems,^9, 10^ less cooperation^11^ and poorer social skills.^9^ Likewise, influences of poor maternal parenting behaviors are comparable to those of postnatal maternal depression and include negative emotionality,^12^ dysregulation of the stress responses,^13, 14^ and poorer child behavior and socio-emotional function.^15, 16, 17, 18, 19, 20, 21, 22^ Both maternal depression and parenting behaviors shape emotional and cognitive information, which affects child development in attention and memory as well as emotional reactivity of the central nervous system.^23^ Nevertheless, limited research has investigated whether both independently or interactively influence child brain development, especially for brain regions associated with emotion.

Associations of maternal depression and poor parenting with the aforementioned child outcomes are likely multifactorial. One possibility is that both maternal depression and poor parenting behaviors can influence children through separate behavioral approach and withdrawal systems. Depressive mothers who display a withdrawn style of interacting, express less positive affect and are more disengaged than non-depressed mothers.^24^ Depressed mothers who display an intrusive style, tend to overstimulate their infants by poking, restraining or aggressively introducing or withdrawing toys.^25^ These behaviors, in which depressed mothers fail to respond appropriately to infant emotional cues or to provide adequate levels of positive affect, may interfere with infant emotional development.^26, 27, 28^ After repeated failure to engage with their mothers, infants may withdraw from interactions with them. Infants may also use less mature regulatory strategies to cope with negative emotions, resulting in them experiencing more negative affect.^29^ Notably, these experiences in early life may lead to the engagement of the neural systems supporting withdrawal.^30^ On the other hand, one facet of parenting behaviors that can be assessed is maternal sensitivity, referring to the ability of a mother to perceive signals from her child and to interpret them accurately and respond promptly and appropriately.^31^ Maternal sensitivity is found to predict a variety of childhood socio-emotional constructs.^15, 16, 17, 18, 19, 20, 21, 22^ This includes attachment security, a construct reflective of whether infants manage distress by approaching or avoiding their mothers.^32, 33^ Maternal sensitivity, may also be expected to influence offspring affective experience, as well as their approach-avoid patterns, which may shape the neural systems supporting approach.

Electroencephalogram (EEG) has been widely used to understand brain function in infants and children. EEG asymmetry reflects the difference between the EEG power observed at the right hemisphere and the left hemisphere.^34^ Convergent evidence shows that left frontal asymmetry of activation is associated with trait tendencies toward a general approach, or behavioral activation motivational system or positive emotion, while right frontal asymmetry of activation is associated with trait tendencies toward a general avoidance or withdrawal system, or negative emotion.^35, 36, 37^ Indeed, right frontal asymmetry of neural activity has been found to be linked with behavioral inhibition and social withdrawal in infants, children and adults.^38, 39^ Likewise, infants of depressed mothers show greater relative right frontal asymmetry compared to infants of non-depressed mothers.^40, 41, 42, 43^ A meta-analysis confirmed right frontal EEG asymmetry in children as a marker of the presence of familial stressors across a wide range of samples.^44^ Patterns of frontal EEG are proposed to serve as markers for the incidence of mood disorders in children^45^ and to predict behavioral and emotional problems.^46^ Moreover, reduced maternal sensitivity is associated with greater relative right frontal asymmetry.^47^

Although many studies show that depressed women display poor parenting, not all depressed mothers are necessarily ineffective in their parenting. For example, Zahn-Waxler *et al.*^48^ find that maternal parenting behavior moderated the association between maternal depression and child behavior problems. Depressed mothers who use proactive childrearing approaches have children who show fewer externalizing problems compared to those whose mothers use more negative reactive approaches. Given such findings, one question of interest is whether maternal sensitivity moderates the effects of maternal depression on infant frontal EEG asymmetry.

In addition, while to date, a large portion of literature focuses on maternal depression or maternal sensitivity and their relations with child outcomes, few studies investigate the time that mothers spend with their children. This oversight may be partly due to the longstanding notion that mothers serve as primary caregiver during infancy.^49, 50^ Moreover, cross-species data suggest that infant expectations about the world are initially shaped by exposure to variations in maternal behavior.^51^ However, the degree to which mothers are present with infants may vary by generation, culture norms,^52^ socioeconomic conditions^53, 54^ and maternal employment status.^55, 56^ For example, time diaries of large samples show that the average time employed women spend caring for their children is less than non-employed women.^55, 56^ Furthermore, in many Asian cultures, it is common for infants to live in the same household as their co-residential grandparents or to be extensively cared for by their grandparents.^57, 58^ Key to the aforementioned argument is the possibility that exposure to *frequent* non-optimal care may solidify into trait-like differences in affect, motivation, and, so, frontal EEG asymmetry. A large portion of mothers in Southeast Asian countries are employed (in our sample, 73.1% of mothers are working). Hence, it is important to consider the amount of time infants spent with their mothers when investigating the influence of exposure to non-optimal caregiving on infant frontal function.

The present study aimed to examine the effects of maternal sensitivity and postnatal maternal depression on frontal EEG asymmetry of offspring at 6 months of age, while considering the amount of time the infants spend with their mothers. Substantial literature has suggested the relationship of maternal depression and right frontal EEG asymmetry of infants and relatively fewer studies investigate the association between maternal sensitivity and frontal EEG asymmetry of infants. However, no studies to date have explored the possible moderation of the relation between maternal depression and infant frontal EEG asymmetry by maternal sensitivity. We were interested in exploring whether exposure to postnatal depression and low maternal sensitivity were independently sufficient to predict right frontal EEG asymmetry, or whether variation in one of these maternal variables might enhance, or buffer against, the other. Hence, in this study, we used data from a longitudinal birth cohort based in Singapore to examine whether maternal depression and maternal sensitivity operate interactively or independently to influence frontal EEG asymmetry. In addition, this cohort study capitalizes on a ‘natural experiment' occurring within the Singaporean caregiving context, allowing us to examine the degree to which time spent with biological mothers influences the aforementioned relations. We hypothesized that postnatal maternal depression and maternal sensitivity influence infant frontal EEG asymmetry only when the time infants spent with their mother is above a certain amount. Finally, given the fact on the association between right frontal EEG asymmetry and negative emotion, we examined whether such an association was also observed in our sample.

## Materials and methods

### Participants

Participants were recruited from Growing Up in Singapore Towards Healthy Outcomes (GUSTO), a longitudinal, Singaporean birth cohort study.^59^ The GUSTO cohort consisted of pregnant Asian women attending the first trimester prenatal ultrasound scan clinic at the National University Hospital (NUH) and KK Women's and Children's Hospital (KKH) in Singapore. The parents were Singapore citizens or Permanent Residents of Chinese, Malay or Indian ethnic background. The GUSTO cohort study was approved by the National Healthcare Group Domain Specific Review Board (NHG DSRB) and the Sing Health Centralized Institutional Review Board (CIRB). In addition, written consent was obtained from mothers.

Socioeconomic status (household income) was extracted from survey questionnaires conducted as part of a scheduled appointment during pregnancy. Birth outcome and pregnancy measures were obtained from hospital record. The present study included healthy term-born infants with gestational age ⩾37 weeks, birth weight ⩾2.5 kg, and a 5-min neonatal Appearance, Pulse, Grimace, Activity and Respiration (APGAR) score ⩾9. These inclusion criteria were applied to limit the influence of variation in maturational effects on the fetal brain development. The full sample of the current study included 111 infants who fulfilled the above inclusion criteria and had good EEG, maternal sensitivity data and maternal depression questionnaire data.

### Maternal depression

The Edinburgh Postnatal Depression Scale (EPDS) questionnaire was administered to mothers at 26 weeks of gestation and 3 months after delivery, and was used to quantify prenatal and early postnatal levels of maternal depressive symptomatology. The EPDS is a widely used 10-item self-report scale designed as a screening instrument for postnatal depression and has been well validated for use in prenatal and postnatal depression.^60^ Each item in the EPDS is scored on a four-point scale (0–3) and items 3 and 5 to 10 are reverse scored. Higher scores indicate a greater intensity of depressive symptoms. The reliability of the EPDS scores assessed using Cronbach's analysis was 0.85 and 0.82 for the prenatal and postnatal EPDS for our cohort, respectively.

### Maternal sensitivity

A 15-min mother–child interaction was recorded as part of a 3-h laboratory visit when infants were 6 months of age (±2 weeks). The mother was asked to ‘interact or play' with her 6-month old infant ‘as she normally would at home'. The one-way mirrored room was equipped with a foldable chair, highchair and a mat, but no toys for the first 5 min. After 5 min, a standard set of attractive toys and books was brought into the room. Maternal sensitivity was assessed using the Revised Mini-A short form of the Maternal Behavioral Q-Sort-V (Mini-MBQS-V).^61, 62^ The Mini-MBQS-V consists of 25 items, each representing different possible aspects of sensitive and insensitive maternal behavior during interaction with an infant. The two Southeast Asian coders who scored the majority of the current study's cases were directly trained by the developers of the Mini-MBQS-V coding system (D. Pederson and S. Bento). The local coders were fluent in both English and the predominant mother tongue languages of Singapore, with one coder fluent in both English and Tamil, and the other in English, Malay and Mandarin. The two local coders achieved a high inter-rater correlation (*r*=0.86) on roughly 15% (*n*=64) of coded cases from the larger GUSTO sample (*n*=424 coded to date).

### Time infants spent with mothers

A self-administered questionnaire capturing information on caregiver involvement and infants' sleeping pattern was given to mothers when infants were 6 months of age (±2 weeks). The mothers were asked to complete the questionnaire based on a ‘typical week' during the past month, first listing all the possible caregivers in relation to the infant. We defined ‘caregivers' as one who spends at least 2 h of time with the infant during the week and is responsible for aspects of the infant's daily routine such as feeding, bathing, playing, reading, changing diapers, cuddling and bedtime routines. Participants were then asked to provide detailed information on the infant's sleeping pattern during a ‘typical week', and to also list the caregivers who were present during the infant's ‘non-nocturnal sleeping hours', that is, hours during the day, inclusive of napping time. If more than one caregiver were listed, participants were then asked to note the one caregiver who is most likely to attend to the infant if he/she is upset or in need during his/her waking hours. We derived the percentage of time infants spent with their mothers during the day, by using the following rationale:


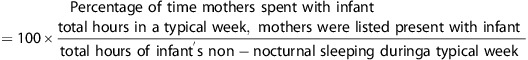


The same rationale was then used to derive the time infants spent with the alternative caregivers (that is, father, maternal grandfather, maternal grandmother, paternal grandfather, paternal grandmother, domestic helper and other caregivers (for example, other relatives or infant care)) by replacing the time infants spent with mothers by the time the infants spent with the alternative caregiver.

### Infant toddler socio-emotional assessment

Mothers reported emotional behavior of their 12-month-old infants using the ‘Infant Toddler Socio-Emotional Assessment' (ITSEA) questionnaire.^63^ The ITSEA is a reliable and validated test for negative emotionality at this age. A higher score on the negative emotionality domain may indicate greater problems with emotion of infants.

### EEG preprocessing and spectral power analysis

The EEG recording procedure was described elsewhere.^64^ Briefly, the 128-channel Geodesic Sensor Nets connected to a DC-coupled amplifier (Net Amp 300, Electrical Geodesic, Eugene, OR, USA) were used to measure EEG signals during the first 2 min of resting state and subsequent 38 min of a passive auditory oddball task in infants at 6 months of age. The first 2-min recording was for the purpose of stabilizing the EEG signal. As it is a common practice in infant research, four eye channels (125, 126, 127, 128) were removed to increase infant tolerance of the procedure. EEG was thus recorded from 124 channels out of 128 channels. At recording, all channels were referenced to vertex Cz position. The signal from all the channels was digitized at 250 Hz and filtered using a band-pass filter with the frequency range of 0.1–100 Hz.

**Figure 1:**
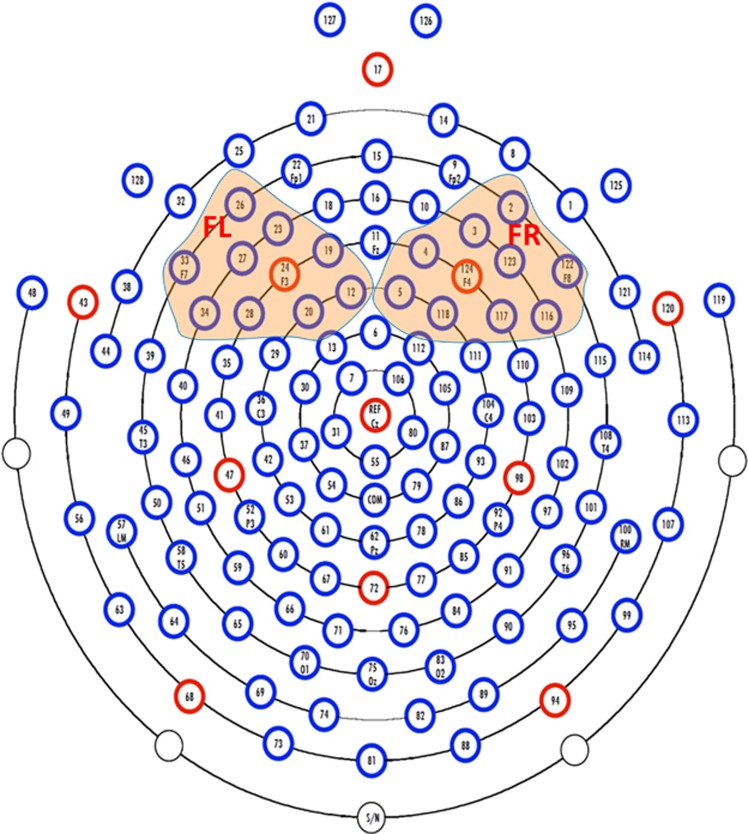
EGI HCGSN128 electrode net. The channels in the left and right frontal regions are highlighted in colored shading. FL, frontal left; FR, frontal right.

For EEG processing, we first removed 25 channels (1, 8, 14, 17, 21, 25, 32, 38, 44, 48, 49, 56, 63, 68, 73, 81, 82, 88, 89, 94, 99, 107, 113, 114, 119), as they are located at the outer ring of the EEG net (Figure 1) and their signals were flat or with motion artifacts. For the rest of the EEG channels (*n*=99), artifacts, including eye blinks and muscle movements, were identified via visual inspection and removed using the EEGLAB toolbox.^65^ Each channel's signal was re-referenced using an average referencing configuration. The mean voltage was subtracted from each channel to eliminate DC effects. Artifacts associated with motor movement over 150 μV peak to peak were also eliminated from all subsequent analyses. Finally, we selected the first 10-min segment of EEG signals after the 2-min resting EEG recording, as this was the longest good continuous EEG segment that can be extracted from all subjects.

The absolute power spectrum of each channel that ranged in the infant alpha frequency band (6–9 Hz) was computed, using a discrete Fourier transform with a Hamming window of a 2s wide epoch and a 50% overlap between epochs. The power spectra were then log-transformed and averaged across the frontal left (FL) channels (12, 19, 20, 23, 24, 26, 27, 28, 33, 34) and frontal right (FR) channels (2, 3, 4, 5, 116, 117, 118, 122,123, 124; see Figure 1). A higher power spectrum value represents lower neural activity.^66^ Frontal asymmetry (FA) power scores were computed as follows, FA=(FR−FL)/(FR+FL). This calculation method has been used in several other studies^67, 68, 69, 70^ to quantify EEG power asymmetry. It has been shown that this asymmetry measure approximately follows Gaussian distribution for the following statistical testing.^71, 72^ A positive FA value reflects greater relative left frontal neural activity than right frontal neural activity (relative left frontal asymmetry), while its negative value reflects greater relative right frontal neural activity than left frontal neural activity (relative right frontal asymmetry).

### Statistical analysis

Multiple regression analyses were used to examine the independent and/or interactive contributions of maternal depression and sensitivity on frontal EEG asymmetry. Postnatal maternal depression and maternal sensitivity as predictors were initially centered to minimize multi-collinearity, and their interaction was formed as the product of the two centered predictors. A hierarchical order of entry was used to enter predictors. Covariates were entered in the first block. The second block included postnatal maternal depression, maternal sensitivity and their interaction. In cases where the interaction term was not significant, a reduced regression model was used to consider independent effects of postnatal maternal depression and maternal sensitivity on frontal EEG asymmetry.

To determine which covariates to enter into the models, we screened the relations between frontal EEG asymmetry and potentially relevant variables (for example, gender, gestational age, birth-weight, prenatal smoking exposure, prenatal alcohol exposure, maternal ethnicity, maternal age at delivery, household income, maternal education and infant age on the EEG visit day (number of days from birth to the visit day)). These variables were considered as potential covariates because of their possible contributions to infant frontal EEG asymmetry. For example, gender differences in frontal EEG asymmetry have been found in infants.^73^ Prenatal smoking exposure and prenatal alcohol exposure were more related to the mother's health. Studies have shown that smoking exposure influences frontal EEG asymmetry in adults.^74^ Maternal ethnicity, maternal age, household income and maternal education were related to socioeconomic status of the infant's family. Given that socioeconomic status was found to be related to adolescent frontal EEG asymmetry,^75^ we examined these variables as potential covariates. In our sample, maternal ethnicity and maternal age were significantly (*P*<0.05) associated with frontal EEG asymmetry and were entered as covariates. In addition, as past research indicated the effects of prenatal maternal depression on frontal EEG asymmetry,^76, 77, 78^ prenatal maternal depression was also entered as a covariate. The whole process led to the inclusion of maternal ethnicity, maternal age and prenatal maternal depression as covariates in the regression analysis mentioned above. Covariates that had a categorical level of measurement (that is, maternal ethnicity) were dummy coded before they were entered into the regression model to ensure their suitability for regression.

**Figure 2:**
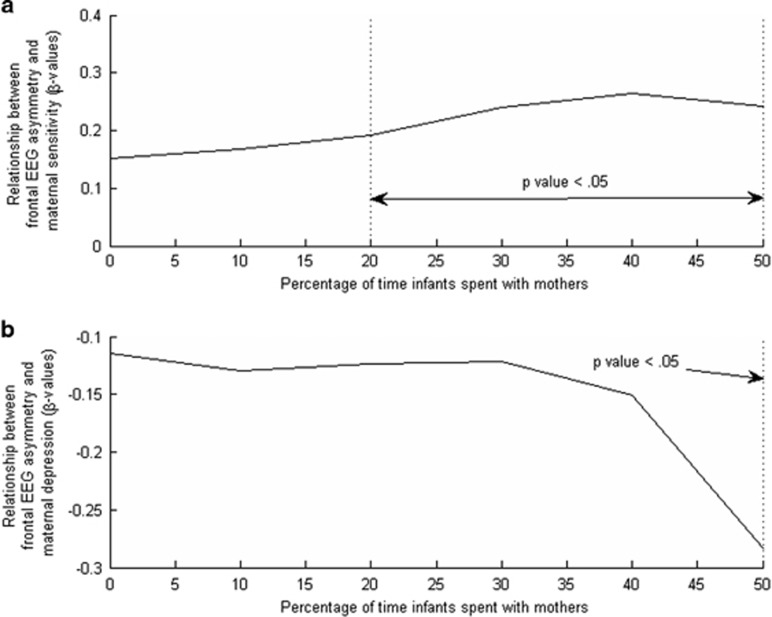
Associations of maternal sensitivity (**a**) and maternal depression (**b**) with infant frontal EEG asymmetry in the subsample with the percentage of time infants spent with their mothers. EEG, electroencephalogram.

Additional regression analysis was performed in the subsample selected based on the amount of time infants spent with their mothers. Since there is no clear cut-off for the mother time spent, we carried out a series of regressions to determine the minimum amount of mother time spent for the predictors (that is, maternal depression and maternal sensitivity) to have an effect on infant frontal EEG asymmetry. These regressions were carried out from at least 0 to 50% mother time spent. Regressions with mother time spent above 50% were not analyzed as the sample size becomes small for statistical power. The results of these series regression analyses (Figure 2) suggested that mothers needed to spend at least 50% of their infants' time for both maternal sensitivity and maternal depression to influence infant frontal EEG asymmetry. Hence, in the subsequent study, infants were split into two subsamples, those who spent at least 50% of their time with their mothers and those who did not.

Following this, regression analyses were performed for two groups of subjects: (1) the full sample and (2) the ‘high mother time spent' subsample. The full sample referred to all the subjects that were included in the study regardless of the amount of time the infant had spent with the mother. The subsample only included infants who had spent at least 50% of their time during their own individualized daytime hours with their mothers.

## Results

### Demographics

Out of 258 GUSTO infants who participated in the EEG recording: 64 infants did not have usable EEG data (for example, data with motion and muscle artifacts); 20 infants did not meet the inclusion criteria; 52 infants whose mothers did not complete questionnaire data (for example, time spent or EPDS questionnaire); and 11 infants whose mothers did not have maternal sensitivity data. Hence, the full sample of the current study included 111 infants. The correlations of maternal age, maternal ethnicity, maternal education, household income and maternal employment status with maternal depression were non-significant (all *P*-values ⩾0.17). Furthermore, among the aforementioned variables, only household income (*r*=0.31, *P*=0.001) and maternal education (*r*=0.25, *P*=0.008) were significantly correlated with maternal sensitivity.

**Figure 3:**
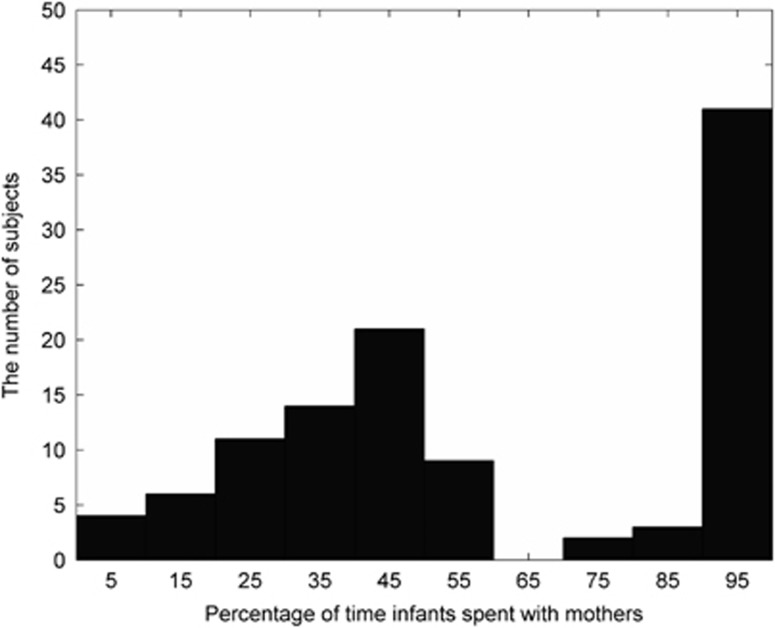
Histogram of the percentage of time infants spent with their mothers.

Figure 3 shows the histogram of percentage of time infants spent with their mothers. The correlation between maternal employment status with mother time spent was significant (*r*=−0.625, *P*<0.001), while maternal age, maternal education and ethnicity were not related to mother time spent (all *P*-values ⩾0.12). Moreover, the correlations between maternal age, maternal education and ethnicity with employment status were not significant (all *P*-values ⩾0.14). However, the correlation between employment status and household income was significant (*r*=0.207, *P* <0.036), suggesting that the mothers who were employed also possessed higher household income.

Among 111 infants, 56 mothers spent <50% of their infants' time and the ‘high mother time spent' subsample consisted of 55 infants. Table 1 lists the demographic information of the full sample (0–100% time spent) as well as the ‘high mother time spent' subsample (51–100% time spent). For each caregiver, time spent with infants is also listed in Table 1. In the full sample and both subsamples, mothers had the highest percentage of time spent among all caregivers. The ‘high mother time spent' subsample did not differ from the ‘low mother time spent' subsample in terms of gestational age, birth weight, AGPAR score, post-conceptual age at EEG visit, frontal EEG asymmetry score, prenatal or postnatal maternal depression score, maternal sensitivity score or maternal age when assessed using independent samples *t*-tests (all *P*-values ⩾0.11). The ‘high mother time spent' subsample also did not differ from the ‘low mother time spent' subsample on categorical variables including gender and prenatal smoking exposure assessed using chi-square tests (all *P*-values ⩾0.51), as well as maternal education, prenatal alcohol exposure and household income, assessed using Fisher's exact test (all *P*-values ⩾0.35). However, there was a significant difference in maternal ethnicity between the ‘high mother time spent' group and ‘low mother time spent' group as assessed using a Fisher's exact test (*P*=0.012).

Among the plausible covariates mentioned in the Materials and Methods section, only maternal ethnicity (*r*=0.223, *P*=0.018) and maternal age (*r*=−0.190, *P*=0.046) were found to significantly correlate with frontal EEG asymmetry. Furthermore, these variables were not found to be correlated to each other. As such, maternal ethnicity and maternal age were added to the analysis as covariates.

### Frontal EEG asymmetry in relation with maternal depression and sensitivity

The results of the regression analysis (Tables 2 and 3) on the full sample showed no significant interaction effect between maternal sensitivity and postnatal maternal depression on frontal EEG asymmetry (*β*=0.061, df=103, *P*=0.49). Likewise, there were no significant effects of maternal sensitivity (*β*=0.152, df=104, *P*=0.10) or postnatal maternal depression (*β*=−0.114, df=104, *P*=0.29) on infant frontal EEG asymmetry.

**Figure 4:**
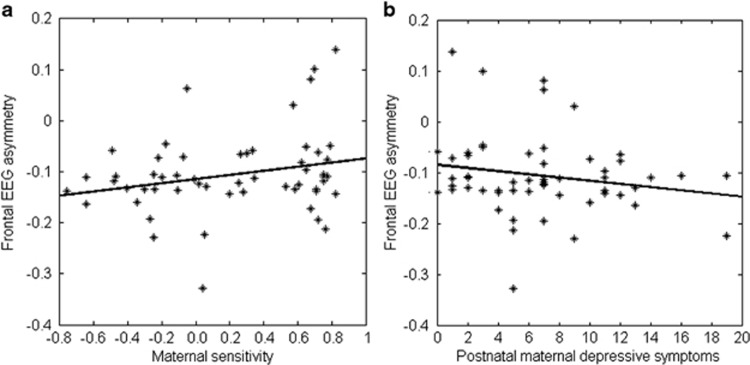
Relations of frontal EEG asymmetry with maternal sensitivity (**a**) and postnatal maternal depressive symptoms (**b**) in the ‘high mother time spent' subsample. EEG, electroencephalogram.

Within the ‘high mother time spent' subsample, there was no significant interaction effect (*β*=−0.082, df=47, *P*=0.52). However, our study revealed that maternal sensitivity (*β*=0.243, df=48, *P*=0.04) and postnatal maternal depression (*β*=−0.283, df=48, *P*=0.04) were significantly associated with frontal EEG asymmetry. These findings (Tables 2 and 3) suggested that lower maternal sensitivity and higher postnatal maternal depression were associated with greater relative right frontal EEG asymmetry. Figure 4 shows the scatter plots of maternal sensitivity on frontal EEG asymmetry and postnatal depression on frontal EEG asymmetry for the ‘high mother time spent' subsample.

### Frontal EEG asymmetry in relation with negative emotionality

Pearson's correlation analysis revealed significant correlation between frontal EEG asymmetry and negative emotionality in the full sample (*r*=−0.255, *P*=0.035, *n*=69), suggesting that greater relative right frontal EEG asymmetry of infants at 6 months of age was associated with greater negative emotionality of infants at 12 months of age. The similar trend was also found in the ‘high mother time spent' subsample (*r*=−0.283, *P*=0.116, *n*=32). This non-significant correlation in the subsample was mainly due to a relatively small sample size even though the correlation coefficient was greater than that from the full sample.

## Discussion

The present study revealed significant associations between both postnatal depression and maternal sensitivity on frontal EEG asymmetry that were apparent only in infants with ‘high mother time spent'. No such effects were apparent in the full sample, where the amount of time that infants spent with their mothers ranged from 0 to 100%. We found no evidence for an interaction effect between postnatal maternal depression and maternal sensitivity in infant EEG frontal asymmetry in both in either the full sample or the ‘high mother time spent' subsample. These findings suggest that for infants who spend at least 50% of time with their mothers, postnatal maternal depression and maternal sensitivity exert similar, but independent effects on frontal EEG asymmetry. That is, among these infants, lower maternal sensitivity and greater maternal depression predicted greater relative right frontal EEG asymmetry. Such greater relative right frontal EEG asymmetry in 6-month-old infants also predicted their greater negative emotionality at 12 months of age

In this study, maternal depression and insensitive parenting did not interactively predict right frontal EEG asymmetry in both the full sample and the ‘high mother time spent' subsample. Our initial hypothesis was motivated by the finding that maternal parenting behaviors could moderate the effect of maternal depression on child behavior problems,^48^ where a large range of parenting behaviors including perspective taking, modulated control and promotion of prosocial approaches, were considered. Maternal sensitivity used in this study constituted only one facet of parenting and variation in sensitivity could be insufficient to moderate the effect of maternal depression on frontal EEG asymmetry.

Similarly, in the full sample, we did not find an independent association of maternal depression or maternal sensitivity with infant frontal EEG asymmetry. Previous studies commonly investigate samples in which mothers were clinically depressed.^40, 79, 80^ Although our sample was a community-based sample, this difference in the depressive scale could partly explain the absence of independent effects between maternal depression and sensitivity on infant frontal EEG asymmetry. An alternative explanation stems from the fact that we utilized an Asian cohort based in Singapore, whereas most other studies utilized Caucasian cohorts.^40, 43^ Given cultural differences in caregiving practices,^52^ it is possible that most of these other studies occurred in a context where mothers were commonly present with their infants, whereas caregiving in our study was more diverse. Mothers in our study may be primary caregivers, however, they may not spend sufficient time with infants for their mood and/or sensitivity to influence their infants' frontal EEG asymmetry.

Notably, in the ‘high mother time spent' subsample both maternal sensitivity and postnatal maternal depression were associated with infant frontal EEG asymmetry and the direction of effects is consistent with a recent meta-analysis examining frontal EEG asymmetry.^44^ For example, Hane and colleagues found lower maternal sensitivity at nine months of age was associated with greater relative right frontal EEG asymmetry during infancy^47^ and at age three.^81^ Similarly, Dawson *et al.*^40^ reported that higher postnatal maternal depression was associated with greater relative right frontal EEG asymmetry.^40^ It is also important to consider that frontal EEG as well as neuroimaging studies reflect the impact of maternal mood across the normal range in shaping neural structure and function.^82, 83, 84^ As expected, our sample suggested that greater relative right frontal EEG asymmetry at 6 months of age predicted greater negative emotionality at 12 months of age. This is largely consistent with the previous finding on the prediction of frontal EEG asymmetry to behavioral and emotional problems.^46^ Hence, the results of this study could highlight the importance of early interventions for improving the quality of care, even in low-risk groups. Infant brain development can be positively impacted from spending considerable time with mothers who are highly sensitive or have low levels of depressive symptoms. This could shift the infant's frontal EEG asymmetry leftwards, which promotes more healthy, positive emotions and approach behavior.^35, 37^ This could then lower the risk for mood disorders^45^ and the likelihood of the infant exhibiting behavioral and emotional problems in childhood.^46^

### Limitations

The study measured maternal sensitivity at 6 months, postnatal maternal depression at 3 months and frontal EEG asymmetry at 6 months. Furthermore, due to the consideration of subject burden, we only assessed prenatal depression at 26 weeks of gestation. Nevertheless, the second and third trimesters of pregnancy are critical periods when neural migration and synaptogenesis of the fetal brain occurs. In addition, our assessment of maternal depression was based on a common screening tool designed to elicit a subjective report of emotional well-being, but which did not constitute a clinical assessment. The reported results are thus best considered as being associated with self-reported depressive symptoms. We report a significant relation between both postnatal maternal depression and maternal sensitivity on infant's frontal function only when infants spent a substantial amount of time with their mothers. However, we cannot exclude the possibility that the limited relationship between these variables observed in the full sample was necessary due to decreased exposure to a depressed or insensitive mothers: such effects might reflect increased exposure to a non-maternal ‘nurturing' caregiver, and thus a protective effect.^85^ Finally, while our data suggest independent influences of postnatal maternal depression and maternal sensitivity, the nature of the effect of postnatal maternal depression remains to be defined and could involve forms of maternal care that are not reflected in the maternal sensitivity measure.

## Conclusion

The present study focused on a community sample of mothers and infants and found that when infants spend a substantial amount of their time with their mothers, both maternal sensitivity and postnatal maternal depression symptomatology associate with infant frontal EEG asymmetry. These effects are consistent with the findings from previous studies such that the lower maternal sensitivity and higher postnatal maternal depression both predict greater relative right frontal EEG asymmetry. These results are interesting to consider in a cross-cultural context. Current practices in many countries substantially influence the amount of time mothers interact with their infants.^52^ In many Asian cultures, it is common for infants to live in the same household as their co-residential grandparents or to be extensively cared for by their grandparents.^57, 58^ Thus, the association of maternal mood or maternal sensitivity on infant's frontal brain function may only be present when infants in Asian cultures spend a considerable amount of time with their mothers.

## Figures and Tables

**Table 1:** Demographics of the full sample, ‘high mother time spent' subsample and ‘low mother time spent' subsample

*Measure*	*Full sample (*N=*111)*	*‘High mother time spent' subsample (*N=*55)*	*‘Low mother time spent' subsample (*N=*56)*
*Infant characteristics*			
Gestational age (week), mean (s.d.)	39.02 (1.01)	39.03 (1.10)	39.02 (0.92)
Birth weight (kg), mean (s.d.)	3.16 (0.38)	3.14 (0.39)	3.18 (0.36)
APGAR, mean	9	9	9
Gender, male/female	49/62	26/29	23/33
Age at EEG visit (week), mean (s.d.)	26.39 (0.95)	26.37 (1.00)	26.40 (0.90)

*Infant frontal EEG*			
Left frontal EEG power, mean (s.d.)	12.90 (2.29)	13.05 (2.44)	12.76 (2.14)
Range	5.22 to 18.09	5.22 to 18.09	7.50 to 17.74
Right frontal EEG power, mean (s.d.)	10.40 (2.20)	10.61 (2.34)	10.21 (2.05)
Range	3.39 to 15.96	3.39 to 15.96	5.86 to 15.18
Frontal EEG asymmetry, mean (s.d.)	−0.11 (0.07)	−0.11 (0.08)	−0.11 (0.06)
Range	−0.33 to 0.14	−0.33 to 0.14	−0.29 to 0.13

*Mother characteristics*			
EPDS Prenatal Maternal Depression score, mean (s.d.)	7.29 (4.42)	7.96 (4.29)	6.63 (4.49)
Range	0.00 to 21.00	0.00 to 21.00	0.00 to 19.00
EPDS Postnatal Maternal Depression score, mean (s.d.)	6.59 (4.75)	6.78 (4.66)	6.41 (4.87)
Range	0.00 to 21.00	0.00 to 19.00	0.00 to 21.00
Maternal sensitivity, mean (s.d.)	0.26 (0.46)	0.21 (0.48)	0.30 (0.44)
Range	−0.76 to 0.90	−0.76 to 0.82	−0.62 to 0.90
Mother time spent (%), mean (s.d.)	61.47 (32.56)	90.40 (17.37)	33.07 (12.89)
Maternal age (year), mean (s.d.)	30.27 (4.71)	30.89 (4.88)	29.66 (4.50)

Maternal ethnicity, %			
Chinese	58.6	54.5	62.5
Malay	28.8	23.6	33.9
Indian	12.6	21.8	3.6

Maternal education, %			
Primary school	2.7	1.9	3.6
Secondary school	18.2	24.1	12.5
Pre-university, diploma or technical course	41.8	35.2	48.2
University undergraduate level	33.6	33.3	33.9
Above university undergraduate level	3.6	5.6	1.8
Prenatal maternal smoking exposure, % yes	39.6	37.0	42.3
Prenatal maternal alcohol exposure, % yes	6.4	5.6	7.3

Maternal employment status, %			
Employed	73.1	48.1	96.4
Unemployed	26.9	51.9	3.6

*Family characteristics*			
Household income (S$), %			
⩽999	2.9	2.0	3.6
1000–1999	9.5	12.0	7.3
2000–3999	30.5	32.0	29.1
4000–5999	26.7	24.0	29.1
⩾6000	30.5	30.0	30.9

*Caregiver*			
Mother time spent (%), mean (s.d.)	61.47 (32.56)	90.40 (17.37)	33.07 (12.89)
Father time spent (%), mean (s.d.)	29.62 (24.37)	29.13 (27.50)	30.10 (21.08)
Maternal grandfather time spent (%), mean (s.d.)	5.87 (20.82)	3.28 (15.23)	8.41 (25.02)
Maternal grandmother time spent (%), mean (s.d.)	13.17 (29.15)	5.01 (17.45)	21.18 (35.61)
Paternal grandfather time spent (%), mean (s.d.)	8.70 (23.18)	8.07 (22.37)	9.33 (24.13)
Paternal grandmother time spent (%), mean (s.d.)	16.27 (32.30)	13.94 (31.34)	18.57 (33.34)
Domestic helper time spent (%), mean (s.d.)	16.99 (32.30)	15.03 (34.89)	18.93 (36.41)
Other caregiver time spent (%), mean (s.d.)	14.59 (27.81)	8.56 (20.86)	20.50 (32.37)

Abbreviations: APGAR, Appearance, Pulse, Grimace, Activity and Respiration; EEG, electroencephalogram; EPDS, Edinburgh Postnatal Depression Scale.

**Table 2:** Interaction effect of maternal sensitivity and postnatal maternal depression on infant frontal EEG asymmetry

*Predictors*	*Full sample*		*‘High mother time spent' subsample*	
	*Δ*R^*2*^	β	*Δ*R^*2*^	β
*Step 1*	0.150**		0.265**	
Covariates				

*Step 2*	0.033		0.122*	
Maternal sensitivity		0.155		0.238*
Maternal depression		−0.115		−0.301*
Interaction		0.061		−0.082

Total *R*^2^	0.182		0.386	
*N*	111		55	

Abbreviation: EEG, electroencephalogram.

Note: *P<0.05 level, **P<0.01 level. Covariates: maternal ethnicity, maternal age and prenatal maternal depression.

**Table 3:** Independent effects of maternal sensitivity and postnatal maternal depression on infant frontal EEG asymmetry

*Predictors*	*Full sample*		*‘High mother time spent' subsample*	
	*Δ*R^*2*^	β	*Δ*R^*2*^	β
*Step 1*	0.150**		0.265**	
Covariates				

*Step 2*	0.029		0.116*	
Maternal sensitivity		0.152		0.243*
Maternal depression		−0.114		−0.283*

Total *R*^2^	0.179		0.381	
*N*	111		55	

Abbreviation: EEG, electroencephalogram.

Note: *P<0.05 level, **P<0.01 level. Covariates: maternal ethnicity, maternal age and prenatal maternal depression.
